# A Massive Number of Extracellular *Tropheryma whipplei* in Infective Endocarditis: A Case Report and Literature Review

**DOI:** 10.3389/fimmu.2022.900589

**Published:** 2022-06-29

**Authors:** Nadji Hannachi, Florent Arregle, Hubert Lepidi, Jean-Pierre Baudoin, Frédérique Gouriet, Hélène Martel, Sandrine Hubert, Benoit Desnues, Alberto Riberi, Jean-Paul Casalta, Gilbert Habib, Laurence Camoin-Jau

**Affiliations:** ^1^Aix Marseille Univ, IRD, APHM, MEPHI, IHU Méditerranée infection, Marseille, France; ^2^Département de Pharmacie, Faculté de Médecine, Université Ferhat Abbas Sétif I, Sétif, Algeria; ^3^IHU Méditerranée Infection, département d'infectiologie, Marseille, France; ^4^Département de cardiologie, la Timone Hospital, AP-HM, Marseille, France; ^5^Laboratoire d’anatomie et de cytologie pathologique, la Timone Hospital, AP-HM, Marseille, France; ^6^Department of Cardiac Surgery, La Timone Hospital, Marseille, France; ^7^Laboratoire d’Hématologie, La Timone Hospital, APHM, Marseille, France

**Keywords:** *Tropheryma whipplei*, infectious endocarditis, rheumatoid arthritis, tocilizumab, electron microscopy

## Abstract

Whipple’s disease (WD) is a chronic multisystemic infection caused by *Tropheryma whipplei*. If this bacterium presents an intracellular localization, associated with rare diseases and without pathognomonic signs, it is often subject to a misunderstanding of its physiopathology, often a misdiagnosis or simply an oversight. Here, we report the case of a patient treated for presumed rheumatoid arthritis. Recently, this patient presented to the hospital with infectious endocarditis. After surgery and histological analysis, we discovered the presence of *T. whipplei*. Electron microscopy allowed us to discover an atypical bacterial organization with a very large number of bacteria present in the extracellular medium in vegetation and valvular tissue. This atypical presentation we report here might be explained by the anti-inflammatory treatment administrated for our patient’s initial diagnosis of rheumatoid arthritis.

## Introduction

Whipple’s disease is a rare multiorgan infective disease caused by an intracellular bacterium, named *Tropheryma whipplei*. Classically, the disease is characterized by weight loss, fever, abdominal pain, diarrhea, polyarthralgia, and lymphadenopathy (1). Infectious endocarditis (IE) caused by *T. whipplei* is rare but represents the most frequent cardiovascular manifestation of *T. whipplei* infection (2). It is now considered to be one of the agents responsible for blood culture-negative endocarditis (3). Analysis of the infected valve reveals vegetations, valvular inflammation, and the presence of *T. whipplei*, mainly observed in monocyte–macrophage cells (4).

We described here a case of a patient treated with anti-interleukin-6, methotrexate, and corticosteroid for his presumed rheumatoid arthritis for 2 years, who was then operated for *T. whipplei* IE. Interestingly, we report particular features in histology and scanning electron microscopy (SEM), notably an abundant presence, mainly in an extracellular environment of *T. whipplei*.

## Case Description

### Patient Presentation

A 69-year-old Caucasian man was referred to our tertiary center for suspicion of native aortic valve IE. He had a history of suspected seronegative rheumatoid arthritis for 2 years with a predominantly rhizomelic localization and was treated with oral corticosteroids, methotrexate, and anti-interleukin-6 (tocilizumab). He was retired from an earthmoving company. The patient was in his usual state of health until 4 days prior to admission when he presented with dizziness. He presented to the emergency department of the nearest hospital where neurologic examination revealed a cerebellar syndrome. The patient had no other symptoms, particularly no fever. Brain MRI showed small ischemic lesions in multiple cerebral territories. Due to a suspected cardioembolic event, transthoracic echocardiography was performed and revealed the presence of significant mobile vegetation on the native aortic valve. The patient was immediately transferred to a tertiary center with cardiac surgery. At admission, his temperature was 37.4°C, blood pressure 112/75 mmHg, heart rate 56 bpm, and oxygen saturation 97%. The patient had no symptoms or clinical signs of heart failure. Neurological examination showed persistence of cerebellar syndrome with dizziness and ataxia of the right upper limb but no motor impairments or sensory disorders. Physical examination revealed no signs of arthritis, but the patient reported morning stiffness in the shoulders and hips. Laboratory tests performed on admission showed a mild inflammatory reaction with a white cell count of 13,000 per microliter and a C-reactive protein level of 7.8 mg/L.

Transesophageal echocardiography was performed and confirmed the presence of a large (16 mm length) mobile vegetation on the aortic valve (left coronary cusp) with moderate aortic regurgitation. There was no sign of aortic root abscess or other valve involvement.

### Microbial Investigations and Treatment

As for each patient presenting in our center with suspicion of IE, we performed a standardized diagnostic test including blood cultures, serological testing for fastidious bacteria, immunological blood tests, and polymerase chain reaction (PCR) from EDTA blood for the detection of bacteria including *Bartonella* species, *Coxiella burnetii*, *Enterococcus faecalis*, *Enterococcus faecium*, *Escherichia coli*, *Staphylococcus aureus*, *Streptococcus gallolyticus*, *Streptococcus oralis*, and *T. whipplei*. Blood test and PCR were performed for *Enterococcus* spp., *E. coli*, and *Staphylococcus* spp. (5). Blood cultures remained sterile and the diagnosis of blood culture-negative IE was made. An antibiotic therapy combining vancomycin and imipenem has been implemented. A cerebral cardiac and thoraco-abdominal CT scan was performed and ruled out other embolic localization. PET-CT did not reveal valvular hypermetabolism. On the fourth day after admission, PCR on blood showed positivity for *T. whipplei*, which was further confirmed on a second sample of blood. The reaction conditions and the primers were previously described by our team (6). PCR for *T. whipplei* in urine and stool was negative but showed positivity from a saliva sample. Antibiotic treatment was changed to an oral combination of doxycycline 100 mg twice daily and hydroxychloroquine 200 mg three times daily for a duration of at least 1 year. Due to the persistence of supracentimetric vegetation and cerebral embolism, an indication of valvular surgery was retained. The patient underwent aortic valve replacement with bioprosthesis 2 weeks after admission. PCR for *T. whipplei* on valvular tissue was positive and pathological examination confirmed the diagnosis of *T. whipplei* IE. Postoperative evolution was simple and the patient was discharged 2 weeks after surgery. The patient had no family antecedents for *T. whipplei* IE.

### Pathological Analysis

A formalin-fixed paraffin-embedded valve sample was cut to 3-μm thickness and stained with hematoxylin–eosin–saffron. Serial sections were also obtained to perform special stains and immunohistochemical investigations. Special stains were used for the detection of bacteria and fungi, including diastase-digested periodic-acid Schiff (PAS), Giemsa, Brown–Hopps/Brown–Brenn Gram, Grocott-Gomori methenamine silver, Warthin-Starry, and Ziehl–Neelsen acid-fast stains. Immunohistochemical analysis was performed with a rabbit anti-*T. whipplei* antibody used at a 1:2,000 dilution, as previously described (7). The immunohistological procedure employed the Ventana Benchmark autostainer (Ventana Medical Systems, Inc., Tucson, AZ, USA). A negative control was performed with normal rabbit serum.

**Figure 1:**
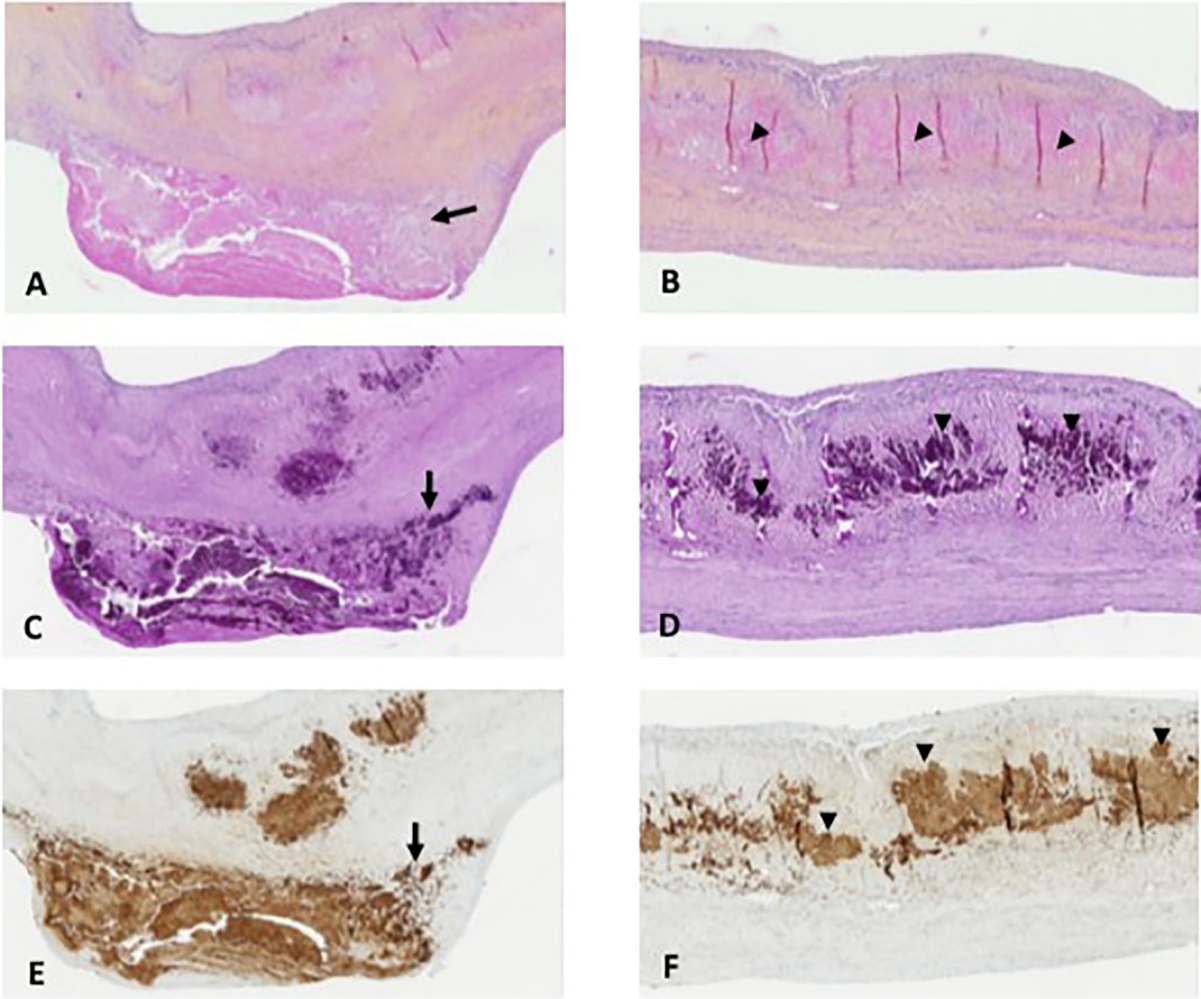
Pathological analysis. **(A, B)** Aortic valve with Whipple’s endocarditis: superficial vegetation and fibrosis of the connective valve tissue and mononuclear inflammatory cell infiltrate with numerous foamy macrophages near the vegetation (**A**, arrow) and with large eosinophilic deposits in the connective valve tissue (arrowheads, **B**) (hematoxylin–eosin–saffron, original magnification ×100 and ×100, respectively). **(C, D)** Foamy macrophages containing the characteristic inclusion bodies (**C**, arrow) and with massive extracellular detection of bacilli with the PAS stain (**D**, arrowheads) (PAS staining, original magnification ×100 and ×100, respectively). **(E, F)** Immunohistochemical detection of *Tropheryma whipplei*: bacilli are packed as coarse granular immunopositive material in foamy macrophage cytoplasm (**E**, arrow) and in an extracellular location in the connective valve tissue in massive number (**F**, arrowheads) (polyclonal rabbit anti-*T. whipplei* antibody used at a dilution of 1:2,000 with Mayer’s hemalum counterstain, original magnification ×100 and ×100, respectively).

*Tropheryma whipplei*-infected aortic valve showed typical histologic features related to infective endocarditis, with vegetation and inflammatory infiltrates in fibrotic tissue (**Figures 1A, B**). Mononuclear cell inflammatory infiltrates were dense and focal, mainly composed of foamy macrophages and lymphocytes. The foamy histiocytes were stuffed with a dense, granular material that was strongly positive on PAS staining and resistant to diastase (**Figure 1C**). Moreover, a massive PAS-positive deposit material was also observed in the valvular connective tissue with an extracellular location (**Figure 1D**). Using the rabbit anti-*T. whipplei* antibody, bacilli were identified in valve tissue by immunostaining. The distribution pattern of immunoreactivity seen with the polyclonal antibody was similar to that observed with the PAS staining. Bacilli were observed as immunopositive material, both in foamy macrophage cytoplasm and in extracellular location (**Figures 1D–F**).

### SEM

SEM analysis was performed on an excised aortic valve consisting in vegetation superposed to a leaflet. As previously described, the valvular tissue was first cut transversally in order to access the internal organization and one-half was further processed for SEM (8). The sample was fixed with glutaraldehyde 2.5% in 0.1 M of sodium cacodylate buffer for 30 min and then rinsed with 0.1 M of sodium cacodylate buffer and distilled water for 1 min each. The sample was dehydrated with increasing ethanol solutions (30%, 50%, 70%, 90%) for 2 min and with 100% ethanol for 5 min. The sample was incubated with ethanol 100%/hexamethyldisilazane (HDMS) 100% in a 1:2 ratio for 5 min. The sample was incubated with 100% of HDMS for 5 min and air-dried for 30 min. It was then mounted with its transverse cut facing upwards on double-sided tape on a clean glass slide and platinum sputter-coated for 40 s at 5 mA (Hitachi MC1000). The observation was made using a SU5000 (Hitachi High-Technologies, Tokyo, Japan) SEM with BSE detector in low-vacuum mode at 15 kV of acceleration voltage, observation mode (spot size 30).

**Figure 2:**
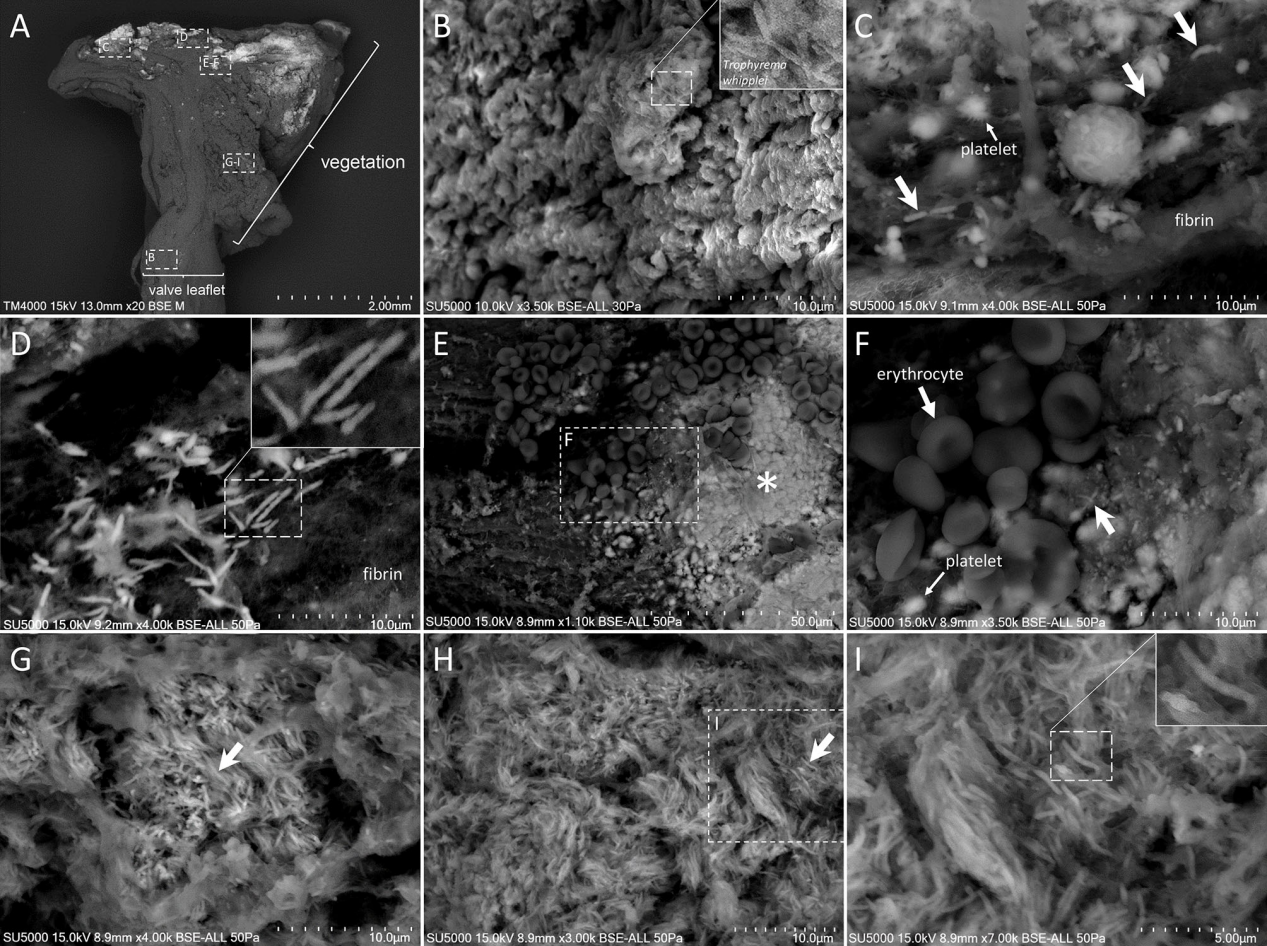
Scanning electron microscopy of *Tropheryma whipplei* vegetation. **(A)** Low-magnification view of the whole cardiac biopsy piece along its deepness, with vegetation (right) superposed to valve leaflet (left). **(B)** Zoom-in image of the [B] boxed region in **(A)** showing a cluster of extracellular *T. whipplei* cells embedded in the valvular tissue. **(C)** Zoom-in image of the **(C)** boxed region in **(A)** showing extracellular *T. whipplei* cells (bold arrows) disseminated in a network of thick fibrin bundles and platelets (thick arrow). **(D)** Zoom-in image of the **(D)** boxed region in **(A)** with extracellular *T. whipplei* cells located in a fishnet-like network of fibrin filaments. **(E, F)** Zoom-in image of the **(E, F)** boxed region in **(A)** containing erythrocytes **(E, F)**, isolated or aggregated platelets (**E, F**, *), fibrin, and extracellular *T. whipplei* cells (arrows). **(G–I)** Zoom-in image of the **(G–I)** boxed region in **(A)** depicting a more central region of the vegetation with numerous *T. whipplei* cells, intermingled with an amorphous matrix and focally organized **(G)** or more broadly distributed **(H, I)**.

SEM analysis was performed on a valvular piece consisting in vegetation anchored to the valve leaflet (**Figure 2A**). Clusters of extracellular bacillus-like objects (200 nm × 2 µm) resembling *T. whipplei* bacteria were found embedded in the leaflet tissue (**Figure 2B**). *Tropheryma whipplei* bacteria were also located extracellularly within the vegetation: i) in the peripheral areas of the vegetation, arranged as single cells disseminated within the other elements of the vegetation (**Figures 2C–F**), and ii) in deeper regions composed exclusively of *T. whipplei* cells, giving the appearance of “vermicelli” (**Figures 2G–I**). In these deep foci, *T. whipplei* cells were densely packed, embedded in an amorphous matrix, and spatially arranged with mixed orientations (**Figures 2G–I**).

## Discussion

We report here a case of a *T. whipplei* IE, with an atypical bacterial organization having a very large number of bacteria present in the extracellular medium in vegetation and valvular tissue. The native aortic valve was recovered from a patient previously treated with a tritherapy consisting of corticosteroid, methotrexate, and anti-IL-6 (tocilizumab) for his pathology presumed to be rheumatoid arthritis. Compared to other Whipple’s endocarditis described in the literature, this case of Whipple’s endocarditis was morphologically similar, with no extensive vegetations, a slight inflammation with inflammatory infiltrates composed mainly of foamy macrophages, and extensive valvular fibrosis (2, 4). In this case, in agreement with previous reports regarding Whipple’s endocarditis, the organisms were found within the cytoplasm of macrophages observed in the inflammatory infiltrates (1, 4). In contrast, massive numbers of bacilli were also detected in an extracellular location at the microscopic and ultrastructural levels, outside of the cytoplasm of macrophages.

An extracellular location of Whipple bacilli was previously described in tissues, but not in such proportions. Indeed, extracellular forms have already been reported in cultures of this species on cell media of fibroblasts in the form of massive aggregates (9, 10). Also, *T. whipplei* was previously grown in an axenic culture medium after sequencing and analysis of its genome to find specific metabolic deficiencies, which allowed the authors to design a complete medium culture (11). The presence of extracellular rRNA specific to *T. whipplei* was described in intestinal biopsies of patients with Whipple’s disease (12). In another study, *T. whipplei* was detected in the glomerular capsular space and in the tubular lumen in a septuagenarian patient treated with etanercept, an anti-TNF-α drug. This localization was objectified by histology and electron microscopy and confirmed by PCR and specific fluorescence *in situ* hybridization (FISH) (13). Also, an extracellular form of *T. whipplei* was reported in a patient with IE who had previously undergone a porcine valve xenograft (14). Here, we provide a description of a massive number of extracellular *T. whipplei*, in a native aortic valve with IE both in vegetation and in the valvular connective tissue.

The epidemiology of *T. whipplei* IE is not perfectly elucidated. Nevertheless, a male predominance is clearly reported, with 85% of the cases being men, mainly European, with an average age of 57.1 years (McGee et al., 2018). Asymptomatic carriage is widely reported in healthy subjects compared to immunodeficient patients, in whom the bacterium may be pathological (15). A recent preliminary study showed that immunocompromised children constituted a reservoir for *T. whipplei* with 77% suffering from acute lymphocytic leukemia (16). In addition, a genetic component cannot be ruled out, since a familial character of the infectious manifestation of *T. whipplei* has previously been reported. Indeed, Guérin et al. described a French family in which four members had Whipple’s disease. These members expressed a rare mutation in one copy of the gene coding for a protein called interferon regulatory factor 4 (IRF4), which acts as a switch that activates and deactivates certain genes involved in the body’s response to infection (17).

This atypical presentation reported here might be explained by the anti-inflammatory treatment administrated for our patient’s initial diagnosis of rheumatoid arthritis. Here, instead of anti-TNF-α, our patient was treated with anti-IL-6 antibodies. Although the explanation of the mechanism of action is far from being elucidated, we may hypothesize that, similar to anti-TNF-α, tocilizumab might have a reactivating effect on the bacteria. In a recent study, confirmed *via* intestinal biopsies and sera of patients, Boumaza et al. reported that anti-TNF-α drugs promote the replication of *T. whipplei* and exacerbate the apoptotic effect exerted by the bacterium on macrophages (18). This might explain the extracellular presence of the bacteria. Anti-TNF-α also allows M2 polarization of macrophages infected with *T. whipplei*, with a less inflammatory and less bactericidal status than M1 macrophages. In the same study, it is noteworthy that TNF-α and interleukin (IL)-6 secretion increased when macrophages were infected with *T. whipplei*, but when anti-TNF-α was added, TNF-α secretion decreased, while IL-6 increased. This observation might suggest a different mechanism of action between TNF-α and IL-6 inhibitors in the reactivation of *T. whipplei* infection (18). In a mouse model evaluating the impact of IL-6 on infection with *Mycobacterium tuberculosis*, an actinobacterium phylogenetically related to *T. whipplei*, lethal tuberculosis was described when mice were IL-6-deficient mutants (19). Numerous studies have already reported cases of Whipple’s disease or infectious endocarditis in patients who received tocilizumab (20–23). However, the involvement of the latter was underestimated, perhaps because it was preceded by anti-TNF-α treatment. Interestingly, in four of the five patients who received tocilizumab in these studies, tocilizumab was the only or the last biologic pro-inflammatory anti-cytokine drug prescribed to them (20–23). In our case, the patient had only received tocilizumab as an anti-cytokine biologic drug, thus emphasizing the possible implication of this therapy with the emergence of *T. whipplei* infection.

In summary, to the best of our knowledge, we report here for the first time a massive number of *T. whipplei* in an extracellular state in clinical isolates. This atypical presentation should draw the attention of clinicians to the risk of pathological expression of the carriage of *T. whipplei* during treatment with anti-IL-6.

## Data Availability Statement

The raw data supporting the conclusions of this article will be made available by the authors, without undue reservation.

## Ethics Statement

Written informed consent was obtained from the individual for the publication of any potentially identifiable images or data included in this article.

## Author Contributions

FA, HL, FG, HM, SH, AR, J-PC, and GH took care of the patient. NH, HL, J-PB, GH, and LC-J did the investigations. NH, FA, HL, GH, and LC-J contributed to the collection of the patient’s medical history. NH, HL, and J-PB provided the figures. The study was designed by NH, FA, GH, and LC-J. Data were interpreted by all authors. The scientific literature search was done by NH, FA, J-PB, BD, GH, and LC-J. NH, FA, HL, J-PB, GH, and LC-J wrote the manuscript. All the other authors commented on and contributed to the final version of the manuscript.

## Conflict of Interest

The authors declare that the research was conducted in the absence of any commercial or financial relationships that could be construed as a potential conflict of interest.

## Publisher’s Note

All claims expressed in this article are solely those of the authors and do not necessarily represent those of their affiliated organizations, or those of the publisher, the editors and the reviewers. Any product that may be evaluated in this article, or claim that may be made by its manufacturer, is not guaranteed or endorsed by the publisher.

## References

[1] Fenollar F Puéchal X Raoult D. Whipple’s Disease. N Engl J Med (2007) 356:55–66. doi: 10.1056/NEJMra062477 17202456

[2] Fenollar F Célard M Lagier JC Lepidi H Fournier PE Raoult D. Tropheryma Whipplei Endocarditis. Emerg Infect Dis (2013) 11:1721–30. doi: 10.3201/eid1911.121356 PMC3837638 24207100

[3] Fournier P-E Gouriet F Casalta JP Lepidi H Chaudet H Thuny F. Blood Culture-Negative Endocarditis: Improving the Diagnostic Yield Using New Diagnostic Tools. Medicine (Baltimore) (2017) 96(47):e8392. doi: 10.1097/MD.0000000000008392 29381916 PMC5708915

[4] Lepidi H Fenollar F Dumler JS Gauduchon V Chalabreysse L Bammert A. Cardiac Valves in Patients With Whipple Endocarditis: Microbiological, Molecular, Quantitative Histologic and Immunohistochemical Studies of 5 Patients. J Infect Dis (2004) 190:935–45. doi: 10.1086/422845 15295699

[5] Habib G Lancellotti P Antunes MJ Bongiorni MG Casalta JP Del Zotti F. 2015 ESC Guidelines for the Management of Infective Endocarditis: The Task Force for the Management of Infective Endocarditis of the European Society of Cardiology (ESC). Endorsed by: European Association for Cardio-Thoracic Surgery (EACTS), the European Association of Nuclear Medicine (EANM). Eur Heart J (2015) 36(44):3075–128. doi: 10.1093/eurheartj/ehv319 26320109

[6] Fournier PE Thuny F Richet H Lepidi H Casalta JP Arzouni JP. Comprehensive Diagnostic Strategy for Blood Culture-Negative Endocarditis: A Prospective Study of 819 New Cases. Clin Infect Dis (2010) 51(2):131–40. doi: 10.1086/653675 20540619

[7] Raoult D La Scola B Lecocq P Lepidi H Fournier PE. Culture and Immunological Detection of Tropheryma Whippelii From the Duodenum of a Patient With Whipple Disease. JAMA (2001) 285:1039–43. doi: 10.1001/jama.285.8.1039 11209175

[8] Hannachi N Lepidi H Fontanini A Takakura T Bou-Khalil J Gouriet F. A Novel Approach for Detecting Unique Variations Among Infectious Bacterial Species in Endocarditic Cardiac Valve Vegetation. Cells (2020) 9(8):1899. doi: 10.3390/cells9081899 PMC7464176 32823780

[9] Marth T. Tropheryma Whipplei, Immunosuppression and Whipple's Disease: From a Low-Pathogenic, Environmental Infectious Organism to a Rare, Multifaceted Inflammatory Complex. Dig Dis (2015) 33(2):190–9. doi: 10.1159/000369538 25925922

[10] Dolmans RA Boel CH Lacle MM Kusters JG. Clinical Manifestations, Treatment, and Diagnosis of *Tropheryma Whipplei* Infections. Clin Microbiol Rev (2017) 30(2):529–55. doi: 10.1128/CMR.00033-16 PMC5355640 28298472

[11] Renesto P Crapoulet N Ogata H La Scola B Vestris G Claverie JM. Genome-Based Design of a Cell-Free Culture Medium for Tropheryma Whipplei. Lancet (2003) 362(9382):447–9. doi: 10.1016/S0140-6736(03)14071-8 12927433

[12] Fredricks DN Relman DA. Localization of Tropheryma Whippelii rRNA in Tissues From Patients With Whipple's Disease. J Infect Dis (2001) 183(8):1229–37. doi: 10.1086/319684 11262205

[13] Moter A Janneck M Wolters M Iking-Konert C Wiessner A Loddenkemper C. Potential Role for Urine Polymerase Chain Reaction in the Diagnosis of Whipple's Disease. Clin Infect Dis (2019) 68(7):1089–97. doi: 10.1093/cid/ciy664 PMC6424077 30351371

[14] Dreier J Szabados F von Herbay A Kröger T Kleesiek K. Tropheryma Whipplei Infection of an Acellular Porcine Heart Valve Bioprosthesis in a Patient Who did Not Have Intestinal Whipple's Disease. J Clin Microbiol (2004) 42(10):4487–93. doi: 10.1128/JCM.42.10.4487-4493.2004 PMC522317 15472298

[15] Boumaza A Ben Azzouz E Arrindell J Lepidi H Mezouar S Desnues B. Whipple's Disease and Tropheryma Whipplei Infections: From Bench to Bedside. Lancet Infect Dis (2022) S1473-3099(22):00128–1. doi: 10.1016/S1473-3099(22)00128-1 35427488

[16] Shams S Niloofar R Beltrame A Moro L Piubelli C Amiri FB. *Tropheryma Whipplei* Intestinal Colonization in Immunocompromised Children in Iran: A Preliminary Study. Future Microbiol (2021) 16:1161–6. doi: 10.2217/fmb-2021-0091 34615382

[17] Guérin A Kerner G Marr N Markle JG Fenollar F Wong N. IRF4 Haploinsufficiency in a Family With Whipple's Disease. eLife (2018) 7:e32340. doi: 10.7554/eLife.32340 29537367 PMC5915175

[18] Boumaza A Mezouar S Bardou M Raoult D Mège JL Desnues B. Tumor Necrosis Factor Inhibitors Exacerbate Whipple's Disease by Reprogramming Macrophage and Inducing Apoptosis. Front Immunol (2021) 12:667357. doi: 10.3389/fimmu.2021.667357 34093562 PMC8173622

[19] Ladel CH Blum C Dreher A Reifenberg K Kopf M Kaufmann SH. Lethal Tuberculosis in Interleukin-6-Deficient Mutant Mice. Infect Immun (1997) 65(11):4843–9. doi: 10.1128/iai.65.11.4843-4849.1997 PMC175695 9353074

[20] Alozie A Zimpfer A Köller K Westphal B Obliers A Erbersdobler A. Arthralgia and Blood Culture-Negative Endocarditis in Middle Age Men Suggest Tropheryma Whipplei Infection: Report of Two Cases and Review of the Literature. BMC Infect Dis (2015) 15:339. doi: 10.1186/s12879-015-1078-6 26282628 PMC4539700

[21] Ramos JM Pasquau F Galipienso N Valero B Navarro A Martinez A. Whipple’s Disease Diagnosed During Anti-Tumor Necrosis Factor Alpha Treatment: Two Case Reports and Review of the Literature. J Med Case Rep (2015) 9:165. doi: 10.1186/s13256-015-0632-6 26215452 PMC4522104

[22] Glaser C Rieg S Wiech T Scholz C Endres D Stich O. Whipple's Disease Mimicking Rheumatoid Arthritis can Cause Misdiagnosis and Treatment Failure. Orphanet J Rare Dis (2017) 12(1):99. doi: 10.1186/s13023-017-0630-4 28545554 PMC5445468

[23] Razanamahery J Humbert S Gil H Bouiller K Magy-Bertrand N. Tropheryma Whipplei Infection Mimicking Giant Cell Arteritis Flare in a Patient Treated With Interleukin-6 Receptor Blocker Tocilizumab. Clin Exp Rheumatol (2020) 38 Suppl 124(2):245–6. 32301419

[24] McGee M Brienesse S Chong B Levendel A Lai K. *Tropheryma Whipplei* Endocarditis: Case Presentation and Review of the Literature. Open Forum Infect Dis (2018) 6(1):ofy330. doi: 10.1093/ofid/ofy330 30648125 PMC6329903

